# Influence of Social Media on the Demand for Esthetic Dental Treatment: Patient Expectations, Risks, and Professional Responsibility

**DOI:** 10.7759/cureus.112928

**Published:** 2026-07-18

**Authors:** Lixmer Rivas Pinto, Pierina Morales Oviedo, Yoley C Paredes Saavedra, Eleazar Garcia Alvarez

**Affiliations:** 1 Dentistry, A.T. Still University, Kirksville, USA; 2 Dentistry, Universidad José Antonio Páez, Valencia, VEN; 3 Dentistry, Universidad Nacional Experimental de los Llanos Centrales Rómulo Gallegos, San Juan de los Morros, VEN; 4 Dentistry, Universidad Central de Venezuela, Caracas, VEN

**Keywords:** body dysmorphic disorder, dental ethics, digital smile design, esthetic dentistry, instagram, patient expectations, social media, tiktok

## Abstract

The pursuit of a perfect smile has become a prominent concern among young adults, and teeth are now read as markers of attractiveness, youth, and social belonging rather than as purely functional structures. Instagram and TikTok sit at the center of this shift, exposing large audiences every day to content on teeth whitening, veneers, smile design, and orthodontics. This narrative review, organized through the Population, Concept, Context framework, examines how these platforms shape esthetic dental trends and patient expectations, and it argues that their value is real but conditional on professional mediation. The trends that circulate most widely include the Hollywood smile, built with porcelain or composite veneers, whitening, esthetic orthodontics, gummy smile correction, and tooth ornamentation. Alongside genuine benefits, wider access to oral health information, stronger patient engagement, and legitimate educational reach, the review identifies unrealistic expectations, misinformation, body image disturbance, demand for irreversible procedures, and an ethical gap around misleading before-and-after imagery and undisclosed sponsorship. The clinician emerges as the decisive variable, since the same platforms that distort expectations can be redirected toward evidence-based education when practitioners manage expectations, screen for body dysmorphic signals, secure informed consent, and uphold advertising ethics.

## Introduction and background

The demand for a perfect smile has gained real weight among young adults. Teeth are no longer judged only by how well they function; they now influence how attractiveness, youthfulness, and social acceptance are perceived, making the smile a factor that carries over into personal and professional life [1]. Against that backdrop, cosmetic dentistry has moved past a purely functional remit and increasingly answers to what patients want their smiles to look like [2].

Over the past decade, the growth of social media has reshaped how people seek information about oral health and cosmetic treatments. Every day, large numbers of users open Instagram, YouTube, and now TikTok to watch content on whitening, veneers, and smile design [3,4]. Unlike conventional media, these platforms let users speak to one another directly, so personal stories and before-and-after photographs end up steering how the public understands cosmetic dentistry [4].

This environment cuts both ways. It has left patients better informed, yet it has also helped normalize unrealistic beauty standards and circulate unreliable advice [5]. Many patients now arrive at the chair with fixed ideas drawn from what they have seen online, which can complicate clinical decision-making and place strain on the relationship between dentist and patient [4,6].

Although research on social media continues to expand, most studies examine a single platform or topic in isolation, leaving the wider picture fragmented. Bringing this evidence together offers a clearer view of how these platforms influence esthetic trends, patient expectations, and everyday practice. This narrative review is guided by the following question: How do social media shape esthetic dental trends and patient expectations, and what benefits, risks, and clinical implications arise for contemporary dental practice?

To address it, the review characterizes the principal esthetic trends driven by social media, weighs their documented benefits against their risks, and examines the clinical and ethical responsibilities they place on the practitioner. The population, concept, and context that frame this review, together with the guiding question, are summarized in Table 1. 

**Table 1:** Population, Concept, Context (PCC) framework and guiding question of the review

Element	Definition in this review
Population (P)	Adults aged 18 and older and members of the general public who use social media to seek or request esthetic dental treatment; secondarily, dental professionals as creators of esthetic content.
Concept (C)	The influence of social media on esthetic dental trends, patient expectations, body image, and treatment-seeking behavior.
Context (C)	The contemporary digital environment, specifically Instagram and TikTok, and its intersection with esthetic dental practice.
Guiding question	How do social media shape esthetic dental trends and patient expectations, and what benefits, risks, and clinical implications arise for contemporary dental practice?

## Review

Methodology

This work is a narrative literature review and does not follow a systematic protocol. It aims to examine and synthesize the available evidence on how social media, particularly Instagram and TikTok, shape esthetic dental trends, influence how people perceive their own appearance, and affect decisions to seek cosmetic dental treatment. The role of dental professionals as creators and distributors of esthetic content is considered alongside that of patients and lay users. No Preferred Reporting Items for Systematic Reviews and Meta-Analyses (PRISMA) guidelines were applied, and no quantitative or statistical synthesis was performed, so the findings are not intended to be statistically generalizable to broader populations.

The Population, Concept, Context (PCC) framework guided the search and defined the scope, since it suits scoping and narrative work. The population comprises adults aged 18 and older and members of the general public who use social media to look for or request esthetic dental treatment. Studies such as those by Abbasi et al. [7] and Baik et al. [8] surveyed exactly these groups, along with general practitioners, to gauge how social media consumption shapes demand. Dentists themselves are also part of the population, given their role as active content creators [7]. The concept is the influence social media exerts on esthetic trends, expectations, body image, and treatment-seeking behavior. Hermans et al. [9] showed that both passive and active use of visually driven platforms is linked to a greater intention to pursue cosmetic procedures and to the normalization of esthetic enhancement among young adults, and Freire et al. [10] found that close to a third of patients who had undergone esthetic dental treatment reported that social media influenced that decision. Mironica et al. [11] add a broader context, identifying Instagram and TikTok among the platforms most responsible for driving esthetic trends across health-related fields. The context is the contemporary digital setting, specifically Instagram and TikTok, and their intersection with esthetic dental practice. Baik et al. [8] reported that Instagram ranked as the second most-used platform among patients actively seeking esthetic dental treatment, underscoring its central place here [7,8,10].

The search drew on PubMed/MEDLINE, Scopus, and Google Scholar, complemented by manual cross-referencing of retrieved articles, and covered the period from 2018 to 2025. Search terms combined English and Spanish keywords, including social media and dental esthetics; Instagram or TikTok and cosmetic dentistry; social media and dental treatment-seeking; body image and Instagram and cosmetic procedures; and influencers and esthetic dentistry. Eligible articles addressed esthetic perception, treatment expectations, body image, or dental care-seeking behavior in relation to social media, and focused on adults aged 18 or older, whether patients, general users, or professionals. Studies that focused exclusively on YouTube, Twitter/X, Facebook, or other platforms without reference to Instagram or TikTok were excluded, as were opinion pieces without empirical data and work published outside the 2018 to 2025 window. Systematic reviews with meta-analyses were used only for contextual background rather than as primary sources [11].

Social media landscape in dentistry

Social media has changed how people obtain, share, and respond to health information at a speed that would have been hard to imagine a generation ago. By January 2024, roughly 239 million people in the United States, close to 70 percent of the population, were using social media, and platforms that began as simple networking sites in the late 1990s now shape how people think, act, and even see themselves [12]. What started with Friendster and MySpace has given way to Facebook, Instagram, and TikTok, which sit at the heart of politics, culture, education, shopping, and healthcare alike [12].

Within dentistry, the shift has been especially visible. The move from a largely static, one-way web to interactive, participatory platforms has opened new channels for patient education while raising fresh concerns about the quality of the health content people encounter, a tension that is sharpest among adolescents who increasingly turn to these platforms for health information [13,14]. Dental professionals now use social media to share guidance on oral health, discuss treatment options and materials, signal pricing, and connect with patients and colleagues, with marketing and education as the main drivers [15].

The profession relies on several platforms, each with its own character. Instagram has become a natural home for cosmetic and esthetic content, and its posts serve as a major source of both reliable information and misinformation while noticeably increasing demand for cosmetic procedures [16]. In one cross-sectional study, nearly two-thirds of respondents named Instagram as their preferred platform for dental content, ahead of Facebook, Twitter, LinkedIn, TikTok, and Snapchat, with almost all reporting daily use [13]. Features such as Stories, Reels, and carousel posts lend themselves to before-and-after images, patient testimonials, and behind-the-scenes glimpses of practice life.

Dental content on TikTok is expanding just as quickly. Oral health remains one of the least met care needs among adolescents, and TikTok's reach carries a double edge, capable of educating patients and of spreading misinformation in equal measure. An analysis of #dentist content from the University of California, San Francisco found that informative videos sit alongside inaccurate ones, and that the latter can nudge viewers toward harmful behavior, which is why practitioners are urged to produce accurate, engaging content of their own [17]. Consistent with this demand, a cross-sectional study of 504 participants found that 29.37 percent reported a substantial social media influence on their decision to undergo esthetic dental treatment, with recent recipients more affected than those treated more than two years earlier [13].

Social media-driven esthetic dental trends

Social media has become a driving force behind trends in cosmetic dentistry, shaping how patients think about their options and choose among them. As more people spend time on these platforms, they encounter more content about cosmetic procedures, prompting many to research treatments before they ever set foot in a clinic. Instagram, TikTok, and YouTube stand out as spaces where patients explore options, compare results, and form expectations about how they might look, and the treatments most often associated with this influence are orthodontics, whitening, crowns, and veneers [13].

The platforms do more than inform; they also steer patients toward particular treatments and particular providers. Before-and-after photographs posted by clinics and dentists weigh heavily in decision-making because they offer a visual stand-in for a possible outcome, and factors such as user comments, digital advertising, the polish of a post, and a practitioner's follower count sharpen interest in specific procedures. Experiences shared by influencers and celebrities feed the same appetite, which shows how social media can push expectations and demand in both helpful and unhelpful directions [4].

Much of this appetite gathers around the Hollywood smile, the uniform, bright result built from porcelain or resin veneers. The more these transformations circulate, the more people want them, yet many patients have little sense of when the procedure is genuinely indicated [18]. Studies show that awareness of veneers as a route to a better smile rarely comes with an understanding of when they are needed, how long they last, what can go wrong, or why preserving natural tooth structure matters, all of which the clinician needs to make explicit before treatment begins [18].

Other trends carry clearer risks. Practices such as mewing and the use of fake braces are presented as easy shortcuts to a better smile despite lacking scientific support and carrying potential for harm [19,20], and videos that show treatments without acknowledging their limitations leave viewers with distorted ideas about what a procedure involves [21]. Tooth ornaments such as gems and grills have followed the same path; they can look harmless but may damage teeth and gums when placed incorrectly, and social media tends to spread them faster than it explains their downsides [22]. Less common procedures, including gummy smile correction, have likewise gained visibility, which can raise useful awareness even as the quality of the content varies from one post to the next, and the risks and benefits go unstated [23]. The cumulative effect is that many patients arrive with expectations that outrun what is realistic, and it falls to the clinician to ground the conversation in evidence [7].

Benefits

A balanced reading of the evidence must acknowledge that social media offers genuine benefits for oral health promotion. Immersive tools such as virtual and augmented reality have shown promise for building oral health knowledge, encouraging preventive attitudes, and strengthening patients' sense of self-efficacy, and they point toward evidence-based promotion strategies that depend on close collaboration between clinicians and technology developers [24].

Interventions delivered through social media have improved oral health knowledge and, in some settings, produced modest behavioral gains and clinical benefit. The strength of that evidence remains limited by methodological weaknesses, differences between platforms, and short follow-up, so social media is best understood as a complement to conventional education rather than a replacement, and only when the content is professionally supervised and scientifically accurate [25,26]. Platforms such as WhatsApp, Telegram, and YouTube have been used to support oral health education and self-care, particularly among adolescents and orthodontic patients, though the certainty of these findings is low, and larger, longer randomized trials are still needed [26]. Within schools and similar settings, social media interventions can improve health literacy and prompt behavioral change when integrated into an evidence-based framework and when educators and clinicians remain involved to ensure the messaging is accurate and consistent [27].

Beyond formal education, social media clearly shapes how patients, especially younger ones, perceive and decide on esthetic treatment. Used well, it broadens access to oral health information, raises awareness of available treatments, and supports patient education when qualified professionals produce the content [13]. The absence of any real quality control works against the benefit, which is why the benefit ultimately depends on the accuracy and ethics of what is posted, and why professional oversight and stronger digital health literacy matter so much. Taken together, immersive technologies and social media are promising resources for education and prevention, provided their use is paired with rigorous evaluation and a steady commitment to evidence-based communication and patient safety [25].

Risks and harms

The same reach that makes social media useful also makes it hazardous. Studies that evaluate purely social media-based interventions tend to show moderate to high methodological quality, while those combining digital and traditional approaches are lower in quality, and few meet high standards for blinding and data collection; behavioral interventions are hard to blind, and future work would benefit from validated instruments and standardized protocols [28].

The clearer harms concern appearance and expectation. Visually focused apps such as Instagram and Snapchat exert a strong pull on how people judge their looks and on the demand for cosmetic procedures. Constant exposure to idealized, digitally altered images has been tied to greater body dissatisfaction and to the internalization of unrealistic standards, with adolescents and young adults especially exposed [11]. Beauty standards are not fixed; they shift with culture and media, and the steady spread of filtered, enhanced faces has pushed those standards toward ideals that are difficult to attain. Prolonged exposure of this kind has been linked to altered body image and may feed into body dysmorphic disorder (BDD), complicating both its recognition and its management, while the pursuit of a digitally constructed face can drive demand for unnecessary treatment, overtreatment, and the disappointment that follows when a clinically sound result fails to match an impossible expectation [29].

Misinformation compounds the problem. Across broad health topics, from physical activity to general wellness, platforms such as YouTube carry a considerable share of low-quality or poorly supported content, and the long-term effects of that exposure remain thinly studied [30]. Much health-related material is produced without professional oversight or scientific validation, and the absence of peer review or quality control allows inaccurate, incomplete, or misleading claims to spread widely, leading several investigators to question the reliability of online content as a primary source of patient education [28]. Social media, then, is a powerful but double-edged instrument whose usefulness rises and falls with the quality of the information it carries, and realizing its benefit while containing its harms will take sustained collaboration among clinicians, educators, technology developers, and regulators [28].

Clinical implications

Taken together, the trends and evidence reviewed above begin to answer the guiding question: social media works at once as an instrument of patient education and as a driver of distorted esthetic expectations, and the clinician stands at the point where those two forces meet.

Scoping evidence makes it clear that active patient involvement in decision-making is central to satisfaction and to the perceived success of the esthetic treatment. Communication that goes beyond a verbal explanation helps patients grasp their options, sets realistic expectations, and strengthens the working relationship between clinician and patient, and the growing use of digital tools offers a real chance to improve that exchange [31]. Randomized evidence suggests that Digital Smile Design (DSD) can outperform conventional planning in patient satisfaction and perceived esthetic outcome, since a digital workflow allows patients to see and help shape a proposal before any irreversible step is taken, thereby supporting more realistic expectations and closer coordination between planning and treatment [32]. Three-dimensional and digital systems extend that advantage by improving precision, predictability, and the ability to tailor a plan to the individual, while giving patients a clearer picture of what a procedure can achieve [33].

The same tools carry a caution. A digital simulation is a planning and communication aid, not a guarantee of the final result, because biological, technical, and patient-related variables all bear on the outcome, and patients need to understand a mock-up as a shared reference rather than a promise [33]. Psychology matters here, too. Traits associated with BDD can leave a patient dissatisfied even when the result is clinically sound, which is why assessment should weigh expectations, motivations, and esthetic concerns before any elective procedure begins, and why recognizing warning signs and referring appropriately is part of responsible care [34].

A good esthetic outcome, therefore, rests on more than technical skill. It draws together clinical judgment, appropriate technology, psychological awareness, and honest communication. Robust informed consent, careful expectation management, and the measured use of tools such as DSD are what allow a clinician to decline inappropriate treatment, document the reasoning, and keep decisions aligned with both the evidence and the patient's genuine interests, particularly when a patient arrives holding a stranger's before-and-after as the standard to match.

Ethical and professional considerations

The growing presence of dentists on Instagram and TikTok has blurred the line between clinical care, marketing, and content creation, and the questions this raises reach well beyond treatment outcomes, which is why they deserve to be treated on their own terms.

Professional codes offer a starting point. The American Dental Association's principle of veracity applies to a social media post no less than to a clinical conversation [35-37]. The difficulty is that before-and-after photographs often tell only part of the story, with digital enhancement, favorable lighting, and no mention of how invasive the procedure really was; posting a patient's images without documented informed consent is itself an ethical breach, however well the post performs [36]. This exposes a tension the profession has been slow to face, namely, what happens when the same person is both clinician and content creator. When dentists work with influencers or become influencers themselves, they take on a marketing role that can pull against the duty to put the patient first, and posting aspirational content, making unsupported claims, or accepting undisclosed sponsorship offends both veracity and beneficence [35].

The problem is not confined to clinicians. An analysis of thousands of dental posts on Instagram found that a meaningful share carried false or misleading information, much of it tied to commercial interest, with products promoted, evidence-based care discouraged, and sponsorships left undisclosed [38]. TikTok adds another layer, since its algorithm surfaces health content based on engagement rather than accuracy, so a polished video promoting a cosmetic procedure can reach millions without any professional review [32]. That reach is most concerning where younger audiences are involved, because regular exposure to filtered cosmetic content raises young people's intention to seek procedures and makes those procedures feel normal, even when the images show results that are not realistically achievable [9].

What emerges is a clear gap in accountability. Codes exist, but they are applied to social media only unevenly; sponsorship disclosures are rare, content aimed at younger users goes largely unregulated, and the platforms are built to reward attention rather than accuracy. Treating these questions as a category distinct from clinical implications is what gives the discussion its critical edge, moving past a description of how social media is used toward the harder question of who is responsible for what gets published and for whom it reaches.

Recommendations and future directions

Several practical directions follow from this evidence. At the level of the individual clinician, expectation management belongs at the center of the esthetic consultation. A patient who arrives with a saved before-and-after needs a frank account of what is realistic, what a procedure involves, and what it cannot deliver, and digital planning tools should support that conversation rather than promise an outcome [31,33]. Consent has to be genuinely informed, which means naming risks, alternatives, and the irreversible nature of procedures such as veneer preparation, and being willing to decline treatment that is not clinically justified [7]. Screening for signs of BDD and referring when appropriate protects both the patient and the outcome, since satisfaction in these cases depends on far more than technical quality [29,34].

At the level of the profession, the duty of veracity needs to extend to every post. Before-and-after imagery should be presented honestly, without misleading enhancement, and sponsorships should be disclosed, in line with the ethical principles that already govern practice [35,36,38]. Clearer guidance on digital advertising, together with attention to how algorithm-driven content reaches minors, would help close the accountability gap, and practitioners are well placed to counter misinformation by producing accurate, engaging content of their own [16,17,39]. Efforts to build patients' digital and media literacy should be pursued alongside this work, so that audiences are better equipped to judge what they encounter online.

For research, the gaps are substantial. Much of the current evidence is cross-sectional and platform-specific, and the field lacks longitudinal data on how sustained exposure to esthetic content affects patient outcomes and professional practice over time [39]. Instruments need validation, effects need to be disaggregated by platform, and the pace at which platforms and marketing tactics evolve means that findings can date quickly, all of which argues for a coordinated and continuing research agenda rather than isolated snapshots [30].

## Conclusions

Returning to the question that guided this review, social media shapes aesthetic dental trends and patient expectations by amplifying a narrow, highly visual ideal of the perfect smile and by accelerating demand for treatment that is at times appropriate and at times not. The answer is therefore two-sided, since these platforms educate and distort in equal measure, and the clinician is the variable that decides which effect prevails.

The main outcomes can be stated briefly. The trends that dominate esthetic content are the Hollywood smile through porcelain or composite veneers, whitening, esthetic orthodontics, gummy smile correction, and ornaments such as gems and grills. The benefits are real but conditional, widening access to oral health information, strengthening preventive awareness, and deepening patient engagement, all of which depend on the accuracy of the content. The risks are equally clear, ranging from unrealistic expectations and misinformation to body image disturbance linked with BDD and demand for unnecessary or irreversible procedures. The clinical and ethical response rests with the dentist, who must manage expectations, secure genuinely informed consent, screen for body image disturbance, and uphold honest, professional communication. When kept within these limits, social media can support evidence-based education without compromising patient safety or the integrity of the profession.
